# Paradoxical Reaction to Alprazolam in an Elderly Woman with a History of Anxiety, Mood Disorders, and Hypothyroidism

**DOI:** 10.1155/2016/6748947

**Published:** 2016-03-22

**Authors:** Daniel Kirkpatrick, Tyler Smith, Mitchell Kerfeld, Taylor Ramsdell, Hasnain Sadiq, Arun Sharma

**Affiliations:** ^1^Department of Psychiatry, Creighton University School of Medicine, Omaha, NE 68178, USA; ^2^Creighton University School of Pharmacy & Health Professions, Omaha, NE 68178, USA

## Abstract

With less than 1% of patients who use benzodiazepines being affected, paradoxical responses to benzodiazepines are rare. In this case report, we outline the course of an 80-year-old female who developed a paradoxical response to benzodiazepines. Significant medical and psychiatric history includes anxiety, mood disorder, hypothyroidism, bilateral mastectomy, goiter removal, and triple bypass. The patient presented with mental status changes, anxiety, motor restlessness, and paranoia. Over time, a temporal relationship between the severity of the patient's motor agitation and intake of alprazolam was observed. As doses of alprazolam were decreased, her motor agitation became less severe. In addition to motor agitation, the patient also demonstrated increased aggressiveness, a subjective feeling of restlessness, and increased talkativeness. As her dose of alprazolam decreased, many of the patient's symptoms were observed to decrease. This case report also discusses theories regarding the pathophysiology of paradoxical reactions to benzodiazepines, known risk factors, and appropriate treatment.

## 1. Introduction 

Benzodiazepines are commonly used in the treatment of anxiety, panic attacks, muscle spasms, seizures, agitation, and insomnia. The clinical action of benzodiazepines is mediated by gamma-aminobutyric acid (GABA) type A chloride channels. Benzodiazepines cause increased transmission of chloride ions by increasing the cycling rate of GABA channels. The inhibitory action of benzodiazepines typically causes relaxation, decreases anxiety, and can cause anterograde amnesia. It is estimated that less than 1% of patients experience atypical responses to benzodiazepines [1]. Though rare, the case report literature includes observations of atypical response to nearly every agent in the benzodiazepine family, with intravenous midazolam being the most represented [1–4]. Interestingly, despite an association between risk factors and advanced age, the authors observed more reports of atypical responses in pediatric populations than in geriatric populations [1–5].

Atypical reactions include increased talkativeness, agitation, excessive movement, hostility, psychosis, and feelings of restlessness [1, 6]. The exact cause of paradoxical reactions to benzodiazepines is not well understood; however, several potential mechanisms have been proposed. Benzodiazepines cause cortical inhibition, which may contribute to the violent or agitated behavior experienced in some paradoxical reactions [3, 6, 7]. Benzodiazepines also alter neurotransmitter concentrations, including serotonin [3, 7]. Decreased serotonin transmission in the central nervous system may contribute to agitated behavior [3, 7].

Risk factors for paradoxical reactions include age (with pediatric and geriatric patients being the most represented), genetics, psychological background, and alcohol use [1, 3, 5–7]. In a recent randomized controlled trial, Shin et al. [5] found paradoxical responses to benzodiazepines to be most influenced by the patient's age (with younger patients having more atypical reactions) and the dosage received (with higher doses being more likely to cause a paradoxical response). In a separate randomized controlled trial conducted by Moallemy et al. [6], an increased infusion rate of midazolam was also positively correlated with the development of paradoxical reactions.

## 2. Case Presentation 

### 2.1. Background

The patient is an 80-year-old female with a medical history that includes significant anxiety, mood disorders, hypothyroidism, tremor, unsteady gait, coronary artery disease, and hyperlipidemia. Her surgical history was positive for goiter removal, bilateral mastectomy, hysterectomy, and triple bypass cardiac surgery. She was brought to the hospital by her family due to changes in her mental status, significant anxiety, gait disturbance, and motor restlessness. The patient has a significant family history of dementia and Alzheimer's Disease.

Five years prior to this presentation, the patient had undergone an inpatient course for severe depression and anxiety. As part of this course, she received electroconvulsive therapy (ECT). Her treatment course was very effective and, after a short stay at an assisted living center, she was discharged back home at baseline. Despite the previous success of ECT, the patient and her family decided that they would not give consent for future ECT treatments.

In the week prior to her presentation at the hospital, the patient's dose of alprazolam was increased from 0.5 mg to 1 mg three times daily. She was also taking quetiapine three times daily and sertraline once daily.

### 2.2. Presentation

At the time of her presentation, the patient was very fearful, anxious, and paranoid. She also perseverated on ECT, making frequent allegations that hospital physicians or staff would force her to undergo this treatment. She also presented with significant tremulousness, motor activation, and unsteady gait.

On admission, the laboratory results and studies in Table 1 were obtained (only responses outside the reference range are included).

Based on her presentation in the emergency department, the patient was admitted to a geriatric inpatient psychiatry unit.

Early in her course, the patient's dosage of alprazolam was increased to 1 mg four times daily. Her symptoms were also noted to be “rapidly worsening.” Due to clinical suspicion of delirium, her mental status was rigorously followed up throughout her inpatient course to detect any change in memory, orientation, or onset of hallucinations. It was also noted that her agitation and inability to sit still were “akathisia-like.” Due to concerns over akathisia, the patient's dose of quetiapine was decreased. Additionally, benztropine 2 mg (twice daily) was added. Because of her low blood pressure and slow heart rate, the patient could not be started on propranolol at this time.

Due to worsening motor agitation, the care team sought a neurology consult. Because of falling concerns, the patient was started on one-to-one care. Despite her frailty and age, she repeatedly leapt from her bed or chair and was constantly agitated and in motion. Still suspecting akathisia, her dose of quetiapine was further decreased and her benztropine dose was maintained. The care team also sought a pharmacy consult.

As per pharmacy consult, alprazolam 1 mg was decreased from four times daily to three times daily. Benztropine and sertraline were discontinued at this time. In this consult, the pharmacist mentioned the possibility that this patient's symptoms were the result of a paradoxical response to benzodiazepines.

Due to concerns over signs of psychosis, the patient was briefly started on 0.5 mg of risperidone at bedtime. She continued to be particularly activated, anxious, and restless. Frequently, she would leap out of her chair during conversations. As the patient appeared to be poorly oriented at times, the care team became suspicious of “agitated delirium.” Previously, Montreal Cognitive Assessment (MOCA) testing and interview had largely shown the patient to have stable mental status and sensorium. Haloperidol 2 mg by mouth was provided as needed due to suspicion of agitated delirium. This addition was observed to improve the patient's ability to function normally. The care team continued weaning the patient from alprazolam.

On day 16 of the patient's stay, the laboratory results in Table 2 were obtained (only responses outside the reference range are included).

At this time, the dose of alprazolam had been reduced to 0.5 mg twice daily and the patient was experiencing visible “improvement in restless[ness] and agitation.” With continued tapering of the alprazolam dose, further improvement in restlessness and agitation was noted by the entire medical team. Significantly, she was able to sit through interviews without demonstrating significant motor agitation. The patient's anxiety remained marked; however, it translated less and less into motor agitation. Regardless of other improvements, she still complained of a subjective feeling of restlessness.

By the time alprazolam was completely discontinued, the patient reported being much closer to baseline. She was able to sit still during interview and exam. Discharge planning was begun. The patient remained anxious but had substantial relief from symptoms of motor agitation, subjective feelings of restlessness, and excessive talkativeness.

## 3. Discussion 

Benzodiazepines are common pharmacologic agents prescribed for the treatment of generalized anxiety disorders and panic disorders and are given to induce sedation. The patient was prescribed benzodiazepine based on her history of crippling anxiety. Interestingly, although benzodiazepine administration typically precipitates rapid improvement in anxiety-related symptoms, this patient did not appear to improve after receiving her regular doses of alprazolam.

Atypical symptoms of benzodiazepines include excessive talkativeness, excessive movement, increased emotional release, hostility and rage, and even new-onset psychosis [1, 6]. During her course, the patient demonstrated all of these symptoms. While increased motor restlessness was the most distinctive symptom, she also demonstrated increased emotionality, increased speech output, aggressiveness, and psychosis (for which she was treated with a short course of risperidone).

### 3.1. Pathophysiology

Although the precise pharmacologic mechanism that underlies paradoxical response to benzodiazepines is incompletely understood, researchers have proposed a few possible mechanisms. These mechanisms include altered neurotransmission, suppression of central nervous system (CNS) function, and compensatory responses to benzodiazepine effects.

#### 3.1.1. Altered Neurotransmission

Benzodiazepines are known to act by increasing chloride transmission at GABA receptors. Increased GABA (neuroinhibitory) activity leads to sedation, decreased anxiety, and possible reductions in pain perception. One possible cause of paradoxical responses to benzodiazepines centers around genetic variability in GABA receptors. In fact, multiple allelic forms of the GABA receptor have been identified [1]. Although varied forms of GABA receptors are known to exist, clinically significant differences among different allelic groups have not been definitively established [1, 8]. It is, however, possible that certain allelic forms of GABA receptors respond differently to benzodiazepines. Some studies have also noted a decrease in the concentration of GABA neurotransmitter among those taking benzodiazepines [9]. It is thought that, in response to these agents, total GABA concentrations can become decreased, leading to heightened neural activation [9]. Other studies propose that changes in cholinergic receptors, serotonin, and other neurotransmitters may underlie atypical responses to benzodiazepines [7, 8, 10].

#### 3.1.2. Suppression of CNS Function

Benzodiazepines suppress neural activity by increasing the effect of GABA (inhibitory) receptors. One theory suggests that increased GABA activity can inhibit the activity of the brain's frontal lobe [11]. Decreased frontal lobe activity could translate into erratic behavior, decreased inhibition, rage, excitement, impaired judgment, or decreased impulse control. In other words, benzodiazepines may decrease an individual's ability to control their impulses. Significantly, atypical responses to benzodiazepines have been observed to be more common in those with cortical loss [5, 7, 11].

#### 3.1.3. Compensatory Response

Some researchers have proposed that paradoxical responses to benzodiazepines may be the result of compensatory reactions within the brain. For example, emergent and rebound withdrawal symptoms have been observed to occur between benzodiazepine doses. Similarly, benzodiazepines have been noted to lose effectiveness due to desensitization of receptors. Downregulation of GABA receptors in response to benzodiazepine use could theoretically explain withdrawal-like symptoms, despite intake of therapeutic doses. Interestingly, receptor desensitization is more likely when high-potency, short-acting benzodiazepines (like alprazolam) are used [9].

### 3.2. Risk Factors

While the exact mechanism for paradoxical reactions to benzodiazepines is unknown, certain behaviors and settings are known to be associated with paradoxical reactions. The most significant risk factors for developing an atypical response to benzodiazepines are age, genetic predisposition, significant history of alcohol use, large benzodiazepine doses, and psychiatric or personality disorders [1]. While this patient's genetic risk factors are unknown, this patient's advanced age, large doses of benzodiazepines (maximal dose four times per day plus additional doses as needed), and anxiety-rich psychiatric history place her at increased risk of responding poorly to benzodiazepines. Also, of note, the anticholinergic effects of her other medications could be an additional contributing factor [8].

As mentioned above, cortical thinning and alterations in neurotransmitters are proposed causes of paradoxical reactions [5, 7]. The patient has a significant family history of dementia and Alzheimer's Disease. Between this family history, her advanced age, and observations of decreased cognitive function, it is likely that she has a thinned cerebral cortex. This clinical observation was radiographically confirmed by a head CT that showed decreased cortical mass. The combination of age-related cortical thinning and benzodiazepine-induced inhibition of cortical function could make an atypical response more likely in this patient. Similarly, paradoxical response to benzodiazepines is linked to altered neurotransmitter levels, including serotonin [7, 8]. As this patient has a diagnosis of mood disorders, specifically major depressive disorder, she is likely to have lower-than-normal serotonin levels. Based on the rationale behind existing theories that explain the pathophysiology of paradoxical response to benzodiazepines, this patient is at a significantly elevated risk. It also bears mention that the proposed mechanisms of atypical benzodiazepine reactions are particularly likely in geriatric populations.

### 3.3. Management

Treatment of paradoxical response to benzodiazepines may include supportive administration of physostigmine, flumazenil, and haloperidol [1]. Physostigmine is an acetylcholinesterase inhibitor that crosses the blood-brain barrier and acts to reverse central nervous system depression. Regardless of these effects, physostigmine is thought to improve paradoxical response to benzodiazepines via a nonspecific antiepileptic effect [1]. Flumazenil antagonizes the benzodiazepine receptor and has clinical use in reversing benzodiazepine overdose. In pediatric populations, it has been observed to improve the symptoms of atypical responses to benzodiazepines [1]. During her clinical course, the patient was not treated with physostigmine or flumazenil. Haloperidol, a first-generation antipsychotic, is thought to improve atypical responses to benzodiazepines via action at dopamine receptors. This action has a calming effect on atypical responders to benzodiazepines. The patient was observed to receive clinically significant benefit from haloperidol administration during her clinical course.

In this patient's case, the factor that seems to have been most successful in decreasing motor agitation was a decrease in the dose of alprazolam. The medical record shows a relatively strong temporal relationship between the dose of alprazolam and her motor agitation.

This patient's paradoxical response to benzodiazepines complicated her clinical course and compromised the care team's ability to return her speedily to baseline. While cessation of benzodiazepines seems to have decreased her emotionality, restlessness, and motor agitation, she remained anxious and depressed.

In this patient's case, motor agitation led the care team to investigate a variety of clinical causes that were unrelated to her intake of alprazolam. These alternate diagnoses included akathisia, activated delirium, and anticholinergic side effects of medications. Interestingly, providing the appropriate clinical treatments for akathisia and activated delirium did not durably improve her symptoms; however, decreasing her dose of alprazolam provided visible relief of many of her symptoms.

## 4. Conclusion 

Throughout the course of this patient's treatment for anxiety, increased motor agitation, and depression, we suspected different causes. At various points in the patient's care, we suspected exacerbation of anxiety, akathisia, agitated delirium, and anticholinergic reactions as the cause of her symptoms. Given the temporal relationship between her course and her intake of benzodiazepines, her continued anxiety after resolution of motor agitation, and the presence of significant risk factors, we believe paradoxical response to benzodiazepines to be the most likely cause of this patient's motor agitation, increased aggressiveness, increased talkativeness, and subjective feelings of restlessness. Given that benzodiazepines have the potential to decrease serotonin transmission in the central nervous system, added caution should be exercised when prescribing them for patients with major depressive disorder.

As this paradoxical response to benzodiazepines hindered our ability to achieve our patient's desired results for the inpatient course, we propose that paradoxical reaction to benzodiazepines be considered in the differential diagnosis of increased motor activity, aggressiveness, and subjective restlessness in the setting of geriatric benzodiazepine use.

## Figures and Tables

**Table 1:** 

UA	Notable results
Blood, UA	Small
Leukocyte esterase	Trace
RBC, UA	0–2
Bacteria, UA	0–10

CBC	Notable results

RBC	3.48
Hemoglobin	11.7
Hematocrit	34.6

CMP	Notable results

Glucose	121
AST	9
GFR MDRD Af Amer	80
GFR MDRD non-Af Amer	69

Other tests	Result

EKG	Nonspecific T-wave changes
CT head without contrast	No evidence of mass or intracranial hemorrhage; mild/moderate ischemic change in white matter; mild/moderate cortical atrophy

**Table 2:** 

CMP	Notable result
Glucose	123
GFR MDRD Af Amer	78
GFR MDRD	67

## References

[1] Mancuso C. E., Gabay M., Tanzi M. G. (2004). Paradoxical reactions to benzodiazepines: literature review and treatment options. *Pharmacotherapy*.

[2] McKenzie W. S., Rosenberg M. (2010). Paradoxical reaction following administration of a benzodiazepine. *Journal of Oral and Maxillofacial Surgery*.

[3] Robin C., Trieger N. (2002). Paradoxical reactions to benzodiazepines in intravenous sedation: a report of 2 cases and review of the literature. *Anesthesia Progress*.

[4] Thurston T. A., Williams C. G. A., Foshee S. L. (1996). Reversal of a paradoxical reaction to midazolam with flumazenil. *Anesthesia a& Analgesia*.

[5] Shin Y. H., Kim M. H., Lee J. J. (2013). The effect of midazolam dose and age on the paradoxical midazolam reaction in Korean pediatric patients. *Korean Journal of Anesthesiology*.

[6] Moallemy A., Teshnizi S. H., Mohseni M. (2014). The injection rate of intravenous midazolam significantly influences the occurrence of paradoxical reaction in pediatric patients. *Journal of Research in Medical Sciences*.

[7] Moon Y. E. (2013). Paradoxical reaction to midazolam in children. *Korean Journal of Anesthesiology*.

[8] Gutierrez M. A., Roper J. M., Hahn P. (2001). Paradoxical reactions to benzodiazepines: when to expect the unexpected. *American Journal of Nursing*.

[9] Breggin P. R. (1998). Analysis of adverse behavioral effects of benzodiazepines with a discussion on drawing scientific conclusions from the FDA's spontaneous reporting system. *Journal of Mind and Behavior*.

[10] Van Der Bijl P., Roelofse J. A. (1991). Disinhibitory reactions to benzodiazepines: a review. *Journal of Oral and Maxillofacial Surgery*.

[11] Paton C. (2002). Benzodiazepines and disinhibition: a review. *Psychiatric Bulletin*.

